# Impacto da Concentração de Ácido Úrico Sérico no Risco de Doença Cardiovascular: Um Estudo Coorte Realizado no Norte da China

**DOI:** 10.36660/abc.20200378

**Published:** 2021-07-07

**Authors:** Qian Nie, Xuemei Zhang, Zhihua Hao, Liqin Wang, Huanxin Liu, Chenghao Liu, Zhongli Wang, Guangyao Song

**Affiliations:** 1 Hebei Medical University Department of Internal Medicine Shijiazhuang City China Hebei Medical University - Department of Internal Medicine, Shijiazhuang City - China; 2 Hebei General Hospital Physical Examination Center ShijiazhuangHebei China Hebei General Hospital - Physical Examination Center, Shijiazhuang, Hebei - China; 3 Hebei Medical University School of Public Health Shijiazhuang CityHebei China Hebei Medical University - School of Public Health, Shijiazhuang City, Hebei - China; 4 Hebei General Hospital Hebei Key Laboratory of Metabolic Diseases Shijiazhuang China Hebei General Hospital - Hebei Key Laboratory of Metabolic Diseases, Shijiazhuang - China

**Keywords:** Hiperuricemia, Ácido Úrico, Doenças Cardiovasculares/incidência, Fatores de Risco, Peso Corporal

## Abstract

**Fundamento:**

Os resultados de estudos anteriores sobre a relação entre ácido úrico sérico (AUS) e o risco de doença cardiovascular (DCV) até agora são inconsistentes devido aos fatores de confusão causados por outros fatores de risco cardiovascular conhecidos.

**Objetivos:**

Este estudo tem o objetivo de avaliar a relação entre o AUS e as DCV incidentes em chineses de meia-idade e idosos, que foram estratificados de acordo com o índice de massa corporal (IMC).

**Métodos:**

Recrutamos 5.721 participantes com idades entre 40 e 75 anos que não tinham diagnóstico de DCV na linha de base, e que foram monitorados de 2008 a 2017. Os participantes foram categorizados em quintis de AUS. A regressão de Cox e a análise de sobrevivência de Kaplan-Meier foram utilizadas para comparar a incidência de DCV entre os grupos de AUS. As correlações entre AUS e a incidência de DCV em grupos com IMC e circunferência de cintura (CC) variados também foram analisadas. Um P valor <0,05 foi considerado estatisticamente significativo.

**Resultados:**

Durante um período médio de monitoramento de 7,6 anos, a incidência de DCV aumentou com o AUS (teste de Log-rank p<0,001). Em comparação com o primeiro quintil, as razões de risco padronizadas (intervalos de confiança de 95%) para p desenvolvimento de DCV foram 1,08 (0,78–1,65), 1,17 (0,88–1,77), 1,47 (1,12–2,21), e 1,68 (1,28–2,44) para o segundo, terceiro, quarto e quinto quintis, respectivamente. Essa relação ficou mais clara em participantes com IMC e CC normais. A razão de risco ajustada para cada aumento de 100 μmol/L de AUS foi de 1,13 (intervalo de confiança de 95%: 1,02–1,39) para eventos de DCV.

**Conclusões:**

O AUS alto é um fator de risco de DCV independente em pessoas de meia-idade e idosas do norte da China. Esse efeito é mantido mesmo depois da estratificação de acordo com medidas de magreza/obesidade.

## Introdução

A hiperuricemia tornou-se altamente prevalente nos últimos anos, provavelmente devido a desenvolvimento financeiro rápido e mudanças no estilo de vida.^1 , 2^ Alguns estudos anteriores demonstraram que a alta concentração de ácido úrico sérico (AUS) está associada a incidências mais altas de fatores de risco convencionais de doenças cardiovasculares (DCV), tais como hipertensão, diabetes e aterosclerose.^3 - 5^ É fato notório que as DCV, incluindo doença arterial coronariana, insuficiência cardíaca e acidente vascular cerebral, são as causas mais comuns de morbidade e mortalidade em todo o mundo.^6 , 7^ Nas últimas décadas, houve um aumento acumulado das evidências de uma relação entre hiperuricemia e a ocorrência e o prognóstico de DCV. No estudo cardiológico de Framingham,^8^ um estudo observacional longitudinal com 6.763 participantes, a concentração de AUS não demonstrou ter uma relação causal no desenvolvimento de doença cardíaca coronária, ou a morte resultante dela. Entretanto, outros estudos demonstraram que a hiperuricemia aumenta o risco de doenças cardiovasculares e cerebrovasculares e de mortalidade.^9 - 11^ Em um estudo transversal realizado no nordeste da China,^12^ o AUS foi associado a doença arterial coronariana em mulheres, especialmente nas com idade superior a 80 anos, mas não em homens. Esses achados contraditórios podem ser explicados por fatores de confusão causados por vários outros fatores de risco.

A hiperuricemia é comum na população chinesa, especialmente nas regiões economicamente desenvolvidas.^13^ Portanto, é importante, para a saúde pública, que sejam determinados o status do AUS como fator de risco para DCV, bem como a concentração de AUS que exige a intervenção. Dessa forma, este estudo tem o objetivo de determinar a relação entre a concentração de AUS e o risco de DCV em um coorte de chineses de meia-idade e idosos em um período de 10 anos.

## Métodos

### Padrões éticos

Todos os procedimentos realizados em estudos envolvendo participantes humanos foram feitos em conformidade com os padrões éticos do comitê institucional e/ou nacional de pesquisa, e com a declaração de Helsinki de 1964 e suas emendas posteriores, ou com padrões éticos comparáveis. O estudo foi aprovado pelo comitê de ética do Hospital Geral Hebei (Nº 190106). O termo de consentimento informado foi obtido de todos os participantes incluídos no estudo.

### População do estudo

Foram recrutados participantes entre 40 e 75 anos de idade durante um check-up detalhado no Hospital Geral Hebei, Shijiazhuang, China, em 2008, para este estudo coorte. Esses participantes eram funcionários de empresas, universidades, organizações governamentais locais e hospitais. Durante a investigação de monitoramento até 2017, os participantes repetiram o check-up todos os anos, incluindo um questionário de entrevistas, exames físicos, e coleta de sangue, de forma semelhante ao realizado no levantamento de linha de base. Este estudo foi aprovado pelo Comitê de Ética Humana do Hospital Geral Hebei (Nº 190106). O termo de consentimento informado escrito foi obtido de todos os participantes.

**Figura 1:**
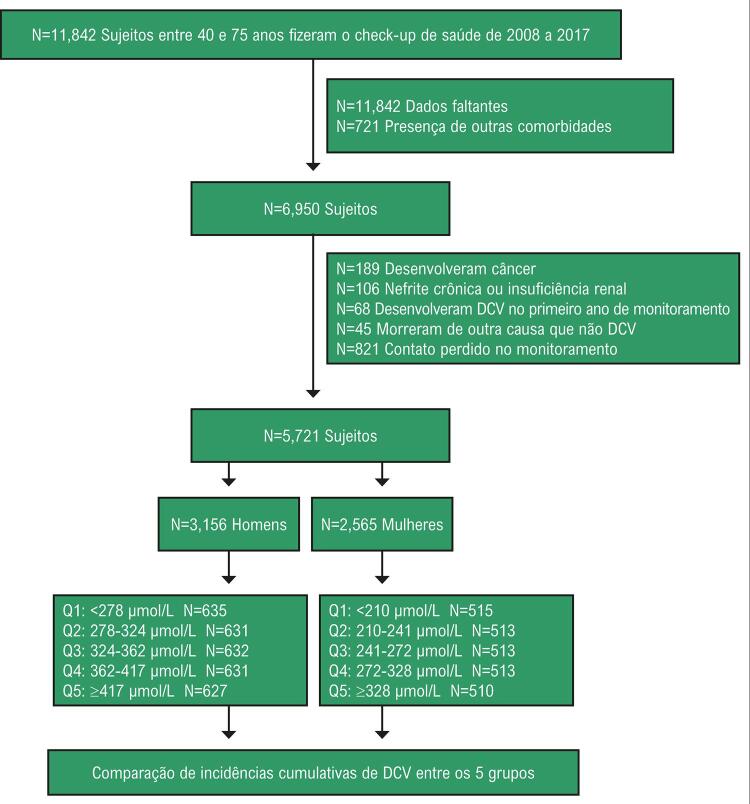
– *Descrição da população do estudo. Os participantes de cada sexo foram alocados separadamente em cinco grupos (Q1 a Q5) com base em sua concentração de AUS.*

Para a análise, DCV foi definida como doença cardíaca isquêmica (DCI), acidente vascular cerebral hemorrágico, ou acidente vascular isquêmico. A DCI foi definida como infarto do miocárdio, angina pectoris, intervenção coronária percutânea, ou cirurgia de bypass da artéria coronária. O coorte incluiu 11.842 participantes que não tinham histórico de DCI, acidente vascular cerebral, câncer, ou insuficiência renal anterior ao período de cadastro, de 2008, e que passaram por exames médicos anuais até 2017. Quatro mil, oitocentos e noventa e dois participantes foram excluídos devido a dados incompletos, presença de comorbidades, ou ausência de uma avaliação do estilo de vida em qualquer um dos anos. Participantes foram excluídos por terem apresentado câncer (n=189), nefrite crônica ou insuficiência renal (n=106), ou um evento de DCV dentro do primeiro ano do estudo (n=68); por terem morrido de outras causas além da DCV (n=45); ou por terem perdido o contato durante o acompanhamento (n=821). Depois da exclusão desses participantes, dados de um total de 5.721 participantes (3.156 homens e 2.565 mulheres) foram analisados ( Figura 1 ).

### Coleta de dados utilizando questionários

Todos os participantes passaram por exames físicos anuais e preencheram um questionário estruturado sobre seu histórico médico geral, uso de medicamentos, histórico de cirurgias, e histórico médico familiar. Questões relacionadas a tabagismo (nunca fumou, ex-fumante ou fumante) e consumo de álcool (nunca bebeu, ex-consumidor e consumidor atual) foram incluídas.

### Medições antropométricas

Os dados antropométricos foram coletados anualmente, incluindo altura, massa corporal, circunferência de cintura (CC), pressão arterial sistólica (PAS), e pressão arterial diastólica (PAD), que foram medidas por enfermeiros bem treinados. O índice de massa corporal (IMC) foi calculado como a massa corporal, em quilogramas, dividida pela altura, em metros, ao quadrado. A pressão arterial foi medida utilizando-se um esfigmomanômetro de mercúrio padrão (Omron HEM-7125, Dalian, China). Os participantes ficaram em repouso por >5 minutos antes da aferição da pressão arterial. A altura e a massa corporal foram medidas com os participantes usando roupas leves e descalços. Essas medições foram feitas duas vezes durante os exames físicos e os valores médios foram utilizados para as análises subsequentes.

### Exames laboratoriais

Amostras de sangue venoso foram coletadas de todos os participantes, após jejum noturno de 12 horas, e foram usadas para medir contagens de leucócitos (WBC) e plaquetas (PLT); concentrações de hemoglobina (Hb), ácido úrico sérico (AUS), glicemia plasmática em jejum (FPG), creatinina (Cr), triglicérides (TG), colesterol total (CT), lipoproteína de baixa densidade (LDL-C), e lipoproteína de alta densidade (HDL-C); e atividades de alanina aminotransferase (ALT) e aspartato aminotransferase (AST). Todas essas medições foram feitas utilizando-se métodos padronizados, com um analisador bioquímico automático (Beckman Coulter AU5800, Tóquio, Japão). A taxa de filtração glomerular estimada (TFG) foi calculada utilizando-se a equação de modificação da dieta em doença renal para pacientes chineses com doença renal crônica.^14^

## Definições

Os participantes de cada sexo foram alocados separadamente em cinco grupos com base em sua concentração de AUS: <278 µmol/L, 278–324 µmol/L, 324–362 µmol/L, 362–417 µmol/L, e ≥417 µmol/L para homens; e <210 µmol/L, 210–241 µmol/L, 241–272 µmol/L, 272–328 µmol/L, ≥ 328 µmol/L para mulheres. A hiperuricemia foi definida como um AUS ≥ 420 μmol/L para homens, e ≥ 360 μmol/L para mulheres.^15^

Hipertensão foi definida como uma PAS ≥ 140 mmHg e/ou PAD ≥ 90 mmHg, ou uso atual de medicamentos anti-hipertensivos.^16^ Diabetes foi definida como um diagnóstico de diabetes feito por um médico, relatado pelo participante, uma FPG ≥ 7,0 mmol/L, ou uso atual de medicamentos antidiabetes.^9^ De acordo com os critérios recomendados para chineses, um IMC de 18,5–23,9 kg/m^2^ foi considerado normal, um IMC de 24,0–27,9 kg/m^2^ foi considerado indicativo de sobrepeso, e um IMC ≥ 28 kg/m^2^ foi considerado indicativo de obesidade. Gordura abdominal foi definida como uma circunferência de cintura (CC) >90 cm nos homens e >85 cm nas mulheres.^17^ Os principais desfechos foram o desenvolvimento de DCI (Classificação Internacional de Doenças-10 códigos I20–25), acidente vascular cerebral hemorrágico (I61), ou acidente vascular isquêmico (I63).

### Análises estatísticas

As características de linha de base dos participantes foram analisadas de acordo com o quintil de AUS, utilizando-se estatísticas descritivas. Variáveis contínuas são expressas como médias ± desvio padrão (DP) e variáveis categóricas, como porcentagens. As variáveis contínuas foram testadas quanto à normalidade por meio de gráficos P-P. Comparações entre dois grupos foram realizadas usando o teste t de Student não pareado para dados normalmente distribuídos, e teste qui-quadrado para dados categóricos. Foram realizadas comparações múltiplas utilizando-se ANOVA de uma via, e o P valor foi corrigido pelo teste post hoc de Bonferroni. A análise de sobrevivência de Kaplan-Meier e o teste de Log-rank foram utilizados para comparar a incidência de DCV entre os grupos de AUS. A regressão de Cox foi utilizada para avaliar a relação entre AUS e a incidência de DCV. As análises estatísticas foram realizadas utilizando-se o software SPSS versão 22.0 (IBM Inc., Armonk, NY, EUA) para Windows. Um p valor <0,05 foi considerado estatisticamente significativo.

## Resultados

### Características de linha de base dos participantes

As características de linha de base dos participantes do coorte, categorizadas de acordo com sexo e quintil de AUS, são apresentadas nas Tabelas 1 e 2 . IMC, CC, PAS, PAD, colesterol total, triglicérides, LDL-C, ALT, Cr, FPG, e a prevalência de consumo de álcool, obesidade, hipertensão e diabetes aumentaram com níveis mais altos de ácido úrico sérico em ambos os sexos. Os homens com os níveis mais altos de concentração de AUS tenderam a ser mais jovens, enquanto as mulheres eram mais velhas. Além disso, uma concentração de AUS alta estava associada a TFG e HDL-C baixos.

**Tabela 1:** – Características de linha de base dos participantes do sexo masculino, categorizadas de acordo com o quintil de concentração de ácido úrico sérico

	Todos	Q1	Q2	Q3	Q4	Q5	p
N (participantes)	3156	635	631	632	631	627	-
AUS (μmol/L)	334,3±40,5	240,2±28,9	296,2±11,8	337,5±13,3	384,4±13,4	456,3±40,7	0,000
Idade (anos)	53,8±9,6	53,2±9,5	52,9±9,6	52,0±9,3	52,4±9,5	51,7±9,4	0,009
IMC (kg/m^2^)	25,6±2,9	24,5±3,2	25,4±2,8	25,7±2,8	26,2±2,7	26,9±2,6	0,000
CC (cm)	89,1±8,3	85,9±8,9	88,3±7,7	89,2±8,2	90,2±7,9	92,0±8,3	0,000
PAS (mmHg)	123,8±16,9	122,2±16,5	122,3±17,0	123,4±16,8	123,0±16,6	124.8±16,7	0,012
PAD (mmHg)	79,5±10,5	78,1±11,3	78,2±10,3	79,8±10,5	80,2±9,8	81,9±10,6	0,004
CT (mmol/L)	4,92±0,85	4,67±0,85	4,76±0,80	4,92±0,83	5,00±0,89	5,12±0,85	0,000
TG (mmol/L)	1,85±0,97	1,55±0,91	1,66±1,01	1,80±1,04	2,06±1,12	2,39±1,23	0,000
HDL-C (mmol/L)	1,27±0,27	1,32±0,28	1,26±0,24	1,25±0,26	1,24±0,25	1,23±0,27	0,000
LDL-C (mmol/L)	2,8±0,77	2,83±0,72	2,85±0,74	2,82±0,82	2,83±0,91	2,86±0,83	0,132
ALT (U/L)	25,0±11,8	23,3±10,5	23,4±10,8	25,4±11,5	28,2±11,3	28,7±11,9	0,000
AST (U/L)	22,7±8,5	22,2±9,3	21,9±6,7	23,1±10,7	23.8±10,2	23,9±6,8	0,097
Cr (μmol/L)	81,0±9,2	75,5±9,8	78,9±9,6	82,4±10,7	83,4±10,6	87,0±11,2	0,000
FPG (mmol/L)	5,96±1,05	5,31±1,81	5,96±0,89	5,97±0,91	6,02±1,11	6,05±0,85	0,000
TFG (ml/min·1,73m^2^)	93,4±12,4	97,8±10,8	95,2±10,9	92,8±11,7	91,8±12,4	87,5±14,8	0,000
Fumantes (%)	40,67	40,39	42,77	41,83	39,98	41,48	0,326
Consumidor atual (%)	37,23	35,69	36,66	38,02	41,86	42,78	0,000
Obesidade (%)	20,8	13,72	16,08	19,44	24,42	31,82	0,000
Hipertensão (%)	16,98	11,76	12,86	14,36	20,16	25,48	0,000
Diabetes (%)	11,08	7,42	8,38	9,79	9,98	13,23	0,000

*AUS: ácido úrico sérico; IMC: índice de massa corporal; CC: circunferência de cintura; PAS: pressão arterial sistólica; PAD pressão arterial diastólica; CT: colesterol total; TG: triglicérides; LDL-C: lipoproteína de baixa densidade-colesterol; HDL-C: lipoproteína de alta densidade-colesterol; ALT: alanina aminotransferase; AST: aspartato aminotransferase; Cr: creatinina; FPG: glicemia plasmática em jejum; TFG: taxa de filtração glomerular estimada. Os dados são expressos como médias ± desvio padrão (DP), se forem contínuos, e como números (porcentagens), se forem categóricos. Foram realizadas comparações utilizando-se o ANOVA de uma via ou o teste qui-quadrado para dados categóricos. As concentrações de ácido úrico sérico para cada quintil foram <278, 278–324, 324–362, 362–417, e ≥417 μmol/L.*

**Tabela 2:** – Características de linha de base dos participantes do sexo feminino, categorizadas de acordo com o quintil de concentração de ácido úrico sérico

	Todos	Q1	Q2	Q3	Q4	Q5	p
N (participantes)	2565	515	513	513	514	510	-
AUS (μmol/L)	259,8±40,9	186,2±18,2	226,0±8,5	255,3±10,9	290,5±13,4	365,2±30,7	0,000
Idade (anos)	52,1±9,0	48,6±7,7	50,3±8,7	52,0±8,6	53,5±8,9	57,1±9,2	0,000
IMC (kg/m^2^)	24,2±3,1	22,8±2,6	23,7±3,0	24,4±3,3	24,7±3,2	25,5±3,1	0,000
CC (cm)	79,3±9,5	75,1±7,2	77,9±8,3	79,6±8,7	81,2±8,6	83,6±7,4	0,000
PAS (mmHg)	118,6±19,3	110,7±15,1	116,7±17,6	118,8±18,5	123,6±19,6	124,0±19,5	0,000
PAD (mmHg)	72,3±9,7	69,1±8,3	71,8±9,8	73,0±9,5	74,2±10,0	74,6±9,8	0,000
CT (mmol/L)	5,15±0,92	4,86±0,90	4,99±0,86	5,21±0,86	5,22±0,93	5,58±0,92	0,000
TG (mmol/L)	1,46±0,95	1,03±0,52	1,22±0,61	1,45±0,59	1,64±0,60	2,09±0,92	0,000
HDL-C (mmol/L）	1,56±0,33	1,61±0,32	1,60±0,31	1,57±0,32	1,50±0,33	1,48±0,36	0,006
LDL-C (mmol/L)	2,92±0,78	2,89±0,73	2,95±0,73	2,99±0,79	3,01±0,81	3,04±0,90	0,056
ALT (U/L)	16,3±10,5	17,9±8,6	17,9±10,3	19,0±8,6	20,6±11,6	21,9±9,3	0,013
AST (U/L)	21,6±10,0	21,3±9,8	20,6±6,9	21,4±7,1	22,3±9,5	22,9±6,0	0,087
Cr (μmol/L)	61,3±9,1	58,6±8,1	59,6±8,4	60,9±8,6	61,9±9,0	66,8±10,02	0,000
FPG (mmol/L)	5,81±1,00	5,61±0,77	5,76±0,91	5,87±1,05	6,00±1,15	6,18±1,00	0,000
TFG (ml/min·1,73m^2^)	97,7±12,3	101,9±11,3	99,9±11,5	96,5±12,6	94,2±12,9	87,1±14,0	0,000
Fumante (%)	4,06	3,72	4,08	3,93	4,70	4,16	0,079
Consumidores atuais (%)	4,73	2,53	5,22	4,01	4,85	6,08	0,000
Obesidade (%)	14,54	3,80	7,23	13,71	16,36	17,51	0,000
Hipertensão (%)	20,34	8,86	16,87	18,39	23,05	26,72	0,000
Diabetes (%)	8,78	2,11	6,02	6,45	10,41	11,30	0,000

*AUS: ácido úrico sérico; IMC: índice de massa corporal; CC: circunferência de cintura; PAS: pressão arterial sistólica; PAD pressão arterial diastólica; CT: colesterol total; TG: triglicérides; LDL-C: lipoproteína de baixa densidade-colesterol; HDL-C: lipoproteína de alta densidade-colesterol; ALT: alanina aminotransferase; AST: aspartato aminotransferase; Cr: creatinina; FPG: glicemia plasmática em jejum; TFG: taxa de filtração glomerular estimada. Os dados são expressos como médias ± desvio padrão (DP), se forem contínuos, e como números (porcentagens), se forem categóricos. Foram realizadas comparações utilizando-se o ANOVA de uma via ou o teste qui-quadrado para dados categóricos. As concentrações de ácido úrico sérico para cada quintil foram <210, 210–241, 241–272, 272–328, e ≥328 μmol/L.*

### Relação entre AUS e o risco de DCV.

**Figura 2:**
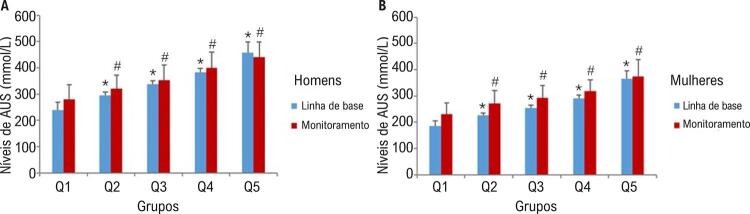
– Concentrações de linha de base e de monitoramento de ácido úrico sérico em participantes de cada um dos sexos. AUS, ácido úrico sérico. Foram realizadas comparações múltiplas utilizando-se ANOVA de uma via, e o p valor foi corrigido pelo teste post hoc de Bonferroni. Tanto em homens (A) quanto em mulheres (B), houve diferenças significativas entre os cinco grupos na linha de base e durante o monitoramento. *p<0,05 (Q1 vs. outros quintis nas concentrações de AUS na linha de base), #p<0,05 (Q1 vs. outros quintis nas concentrações de AUS durante o período de monitoramento).

As concentrações de AUS na linha de base e no monitoramento são comparadas para cada um dos sexos na Figura 2 . O AUS aumentou significativamente do Q1 para o Q5, tanto na linha de base quanto no período de monitoramento, de acordo com o ANOVA. A concentração média de AUS foi mais baixa no final do estudo do que na linha de base nos homens do Q5, mas, ainda assim, atendendo ao critério diagnóstico de hiperuricemia. As características de linha de base dos participantes, categorizadas de acordo com DCV incidente, são apresentadas na Tabela 3 . O período médio de monitoramento foi de 7,6 anos, durante os quais a incidência cumulativa de DCV foi de 14,3% (n=821) entre os participantes, com incidências de 13,9% (n=438) em homens e 14,9% (n=383) em mulheres. Além disso, comparados a participantes que não desenvolveram DCV, os que desenvolveram tinham IMC, CC, PAS, PAD, CT, TG, LDL-C, ALT e FPG mais altos. Não houve diferença significativa entre os dois grupos em relação à Cr.

**Tabela 3:** – Características de linha de base dos participantes do sexo masculino, categorizadas por doença cardiovascular desenvolvida ou não

	DCV (-)	DCV (+)	p
AUS (μmol/L)	299,4±75,1	319,5±73,6	0,012
Idade (anos)	52,2±9,2	59,6±9,1	0,000
Homens (%)	47,1	53,4	–
IMC (kg/m^2^)	24,9±3,1	25,7±3,2	0,001
CC (cm)	84,4±9,7	87,4±9,5	0,000
PAS (mmHg)	120,9±18,1	131,9±19,0	0,000
PAD (mmHg)	76,1±10,7	77,8±10,6	0,037
CT (mmol/L)	5,0±0,8	5,3±0,9	0,001
TG (mmol/L)	1,6±0,7	2,0±1,0	0,000
HDL-C (mmol/L)	1,41±0,3	1,27±0,3	0,000
LDL-C (mmol/L)	2,86±0,7	3,1±0,8	0,034
Cr (μmol/L)	71,8±14,3	72,1±14,1	0,061
FPG (mmol/L)	5,9±1,0	6,4±1,2	0,000
**Tabagismo**			
Nunca (%)	67,3	58,4	
Ex-fumante (%)	11,6	17,7	
Fumante (%)	21,0	23,8	0,000
**Consumo de álcool**			
Nunca (%)	76,1	69,5	
Ex-consumidor (%)	3,2	8,6	
Consumidor atual (%)	20,6	21,8	0,000

*AUS: ácido úrico sérico; IMC: índice de massa corporal; CC: circunferência de cintura; PAS: pressão arterial sistólica; PAD pressão arterial diastólica; CT: colesterol total; TG: triglicérides; LDL-C: lipoproteína de baixa densidade-colesterol; HDL-C: lipoproteína de alta densidade-colesterol; Cr: creatinina; FPG: glicemia plasmática em jejum. Comparações entre os grupos foram realizadas usando o teste t de Student para variáveis contínuas e teste qui-quadrado para dados categóricos. Os dados são expressos como médias ± desvio padrão (DP), se forem variáveis contínuas, e como números (porcentagens), se forem variáveis categóricas.*

**Figura 3:**
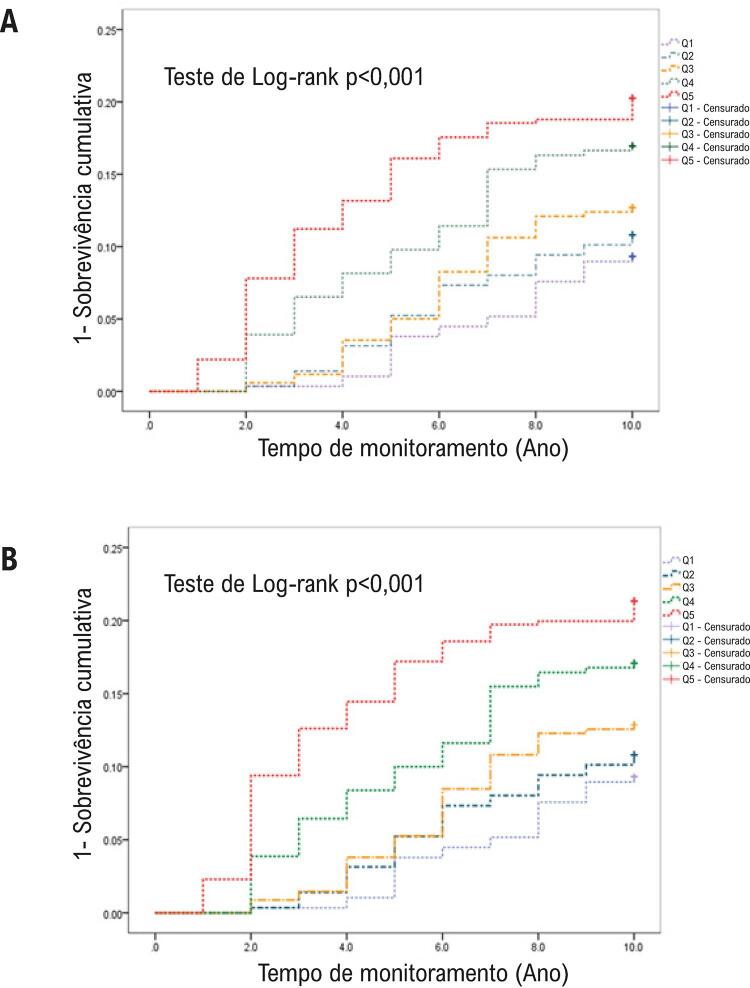
– *Curvas de Kaplan-Meier para doença cardiovascular incidente para cada quintil de concentração de ácido úrico sérico (AUS). (A) Todos os participantes (p-valor >0,01 no teste de Log-rank, Q1 vs. outros quintis). (A) Participantes com TFG normal (p-valor >0,01 no teste de Log-rank, Q1 vs. outros quintis).*

Conforme mostrado na Figura 3A , a análise de Kaplan-Meier da incidência de DCV nos quintis de AUS durante os 10 anos do estudo demonstrou diferenças significativas entre os grupos (teste de Log-rank p <0,001). Os aumentos de AUS foram associados a incidência de DCV mais alta. Como o AUS é afetado pela função renal, em seguida, os participantes foram categorizados de acordo com TFG, e os dados foram analisados novamente. Identificou-se que a relação entre AUS e DCV permaneceu intacta em participantes que tinham valores de TFG dentro da faixa normal (teste de Log-rank p <0,001; Figura 3B ).

A regressão de Cox foi utilizada para avaliar a relação entre a concentração de AUS e a incidência de DCV em mais detalhes. ( Tabela 4 ). No modelo bruto, em comparação com o Q1 de AUS, as razões de risco (RR) para DCV nos outros quatro quintis de AUS foram 1,18 (0,82–1,70), 1,41 (1,01–1,98), 1,95 (1,40–2,71), e 2,58 (1,84–3,61), respectivamente. Os riscos permaneceram significativamente diferentes após a padronização por idade no modelo 2, e por idade e sexo no modelo 3. A RR diminuiu gradualmente à medida que o número de covariáveis padronizadas aumentou, mas a associação entre AUS e DCV permaneceu significativa após a padronização para idade, sexo, TFG, IMC, PA, colesterol total, triglicérides, FPG, tabagismo e consumo de álcool (modelo 4). Em comparação com o Q1, as RR (intervalos de confiança [IC] de 95%) para os quintis Q2–Q5 foram 1,08 (0,78–1,65), 1,17 (0,88–1,77), 1,47 (1,12–2,21), e 1,68 (1,28–2,44). A RR ajustada para cada aumento de 100 μmol/L de AUS foi de 1,13 (IC de 95%: 1,02–1,39) para eventos de DCV. Em seguida, os dados foram analisados novamente após a estratificação por IMC e CC, e identificou-se que a associação significativa entre AUS e DCV ficou mais evidente em participantes com IMC e CC normais ( Tabela 5 ).

**Tabela 4:** – Razões de risco de doença cardiovascular incidente

	Número de eventos	Razão de risco (IC 95%)			
	
Níveis de AUS		Modelo 1	Modelo 2	Modelo 3	Modelo 4
Q1	106/1150	1	1	1	1
Q2	124/1144	1,18(0,82-1,70)	1,56(0,80-1,66)	1,12(0,77-1,63)	1,08(0,78-1,65)
Q3	146/1145	1,41(1,01-1,98)	1,34(0,96-1,89)	1,27(0,91-1,79)	1,17(0,88-1,77)
Q4	203/1145	1,95(1,40-2,71)	1,73(1,25-2,41)	1,61(1,15-2,23)	1,47(1,12-2,21)
Q5	242/1137	2,58(1,84-3,61)	2,08(1,48-2,91)	1,89(1,34-1,34)	1,68(1,28-2,44)
AUS por 100 μmol/L		1,25(1,11-1,42)	1,18(1,04-1,34)	1,16(1,09-1,29)	1,13(1,02-1,39)

*IC: intervalo de confiança; AUS: ácido úrico sérico. O Modelo 1 utilizou dados brutos. O Modelo 2 foi padronizado para idade. O Modelo 3 foi padronizado para idade e sexo. O Modelo 4 foi padronizado para idade, sexo, TFG, índice de massa corporal, pressão arterial, colesterol total, triglicérides, glicemia plasmática em jejum, tabagismo, e consumo de álcool.*

**Tabela 5:** – Razões de risco padronizadas para risco de DCV associada a concentração de ácido úrico sérico, estratificadas de acordo com índice de massa corporal e circunferência de cintura

	Razões de risco (IC 95%)				

	Q1	Q2	Q3	Q4	Q5
IMC (≤23,9)	Referência	1,15(0,92-1,29)	1,21(0,98-1,56)	1,26(1,01-1,64)	1,32(1,08-1,82)
IMC (24,0-27,9)	Referência	1,08(0,78-1,67)	1,13(0,69-1,89)	1,15(0,87-1,65)	1,18(0,98-1,83)
IMC (≥28)	Referência	0,92(0,74-1,56)	1,01(0,85-1,85)	0,98(0,78-1,49)	1,08(0,95-1,77)
CC (homens <90, mulheres <85)	Referência	0,89(0,78-1,45)	1,08(0,88-1,37)	1,12(1,01-1,43)	1,21(1,03-1,59)
CC (homens ≥90, mulheres ≥85)	Referência	1,02(0,72-1,78)	1,15(0,83-1,85)	1,23(0,75-1,91)	1,30(0,91-1,89)

*As razões de risco padronizadas foram calculadas no Modelo 4 e foram estratificadas de acordo com índice de massa corporal (IMC (kg/m^2^) e circunferência de cintura (CC, cm). O Q1 de AUS foi utilizado como grupo de referência. As covariáveis utilizadas no Modelo 4 foram idade, sexo, TFG, índice de massa corporal, pressão arterial, colesterol total, triglicérides, glicemia plasmática em jejum, tabagismo, e consumo de álcool.*

## Discussão

No presente estudo coorte, foi identificada uma relação positiva significativa entre concentração de AUS e a incidência de DCV em chineses de meia-idade e idosos. Essa relação foi independente dos possíveis fatores de confusão de idade, IMC, PAS, PAD, CT, TG, HDL-C, LDL-C, FBG, e histórico de hipertensão ou diabetes. Além disso, demonstrou-se que um incremento de 100 μmol/L de AUS aumenta o risco de DCV em 13%. Esses resultados demonstram que a concentração de AUS é um fator de risco independente para DCV.

Estudos anteriores também demonstraram uma relação entre AUS e DCV em outros países e regiões. Uma meta-análise anterior demonstrou que a hiperuricemia aumenta o risco de DCV, que era 2,1 vezes mais alto em pessoas com AUS alto do que naquelas com AUS baixo.^18^ Outra meta-análise de 16 estudos coorte demonstrou que a hiperuricemia está associada a um risco mais alto de acidente vascular cerebral (razão de risco: 1,41, IC 95%: 1,05–1,76) e mortalidade relacionada a acidente vascular cerebral mais alta (razão de risco: 1,36, IC de 95%: 1,03–1,69), e essas relações se mantiveram significativas após a padronização em relação a outros fatores de risco, tais como, sexo, idade e a presença de hipertensão ou diabetes.^19^ Há também uma quantidade crescente de evidências de que a hiperuricemia aumenta o risco de doenças cardiovasculares e cerebrovasculares, e de mortalidade.^9 , 11 , 20 - 22^ No presente estudo, os participantes era pessoas de meia-idade e idosas que vivem em cidades no norte da China, mas os achados não só são consistentes com achados anteriores, como também vão além deles.

Entretanto, a maioria dos estudos anteriores se concentraram na relação entre AUS e a mortalidade relacionada a DCV, e somente alguns foram voltados para a relação com a incidência de DCV. Vale ressaltar que um estudo coorte de 10 anos com 128.569 pessoas realizado em Taiwan^22^ demonstrou que a hiperuricemia é um fator de risco potencial de DCI. Entretanto, um ponto fraco desse estudo foi o fato de o AUS ter sido dividido apenas entre os grupos de AUS alto e AUS normal, e a relação foi padronizada apenas em relação ao histórico de hipertensão ou diabetes, tabagismo, consumo de álcool, e o uso de diuréticos. Dessa forma, outras covariáveis importantes, tais como o IMC, a idade, e a TFG não foram padronizados. O objetivo do presente estudo foi sanar essas deficiências. Foram utilizados quintis de AUS para demonstrar a relação entre AUS e DCV após a padronização para uma ampla série de fatores de risco convencionais. Além disso, essa relação foi mantida quando o AUS foi tratado como variável contínua. Estudos anteriores também demonstraram uma relação contínua entre o AUS e as DCV,^9^ e, no presente estudo, os valores de AUS no Q4, que não ultrapassaram a faixa de referência, foram associados a um risco de DCV significativamente mais alto.

O mecanismo pelo qual o AUS alto pode aumentar o risco de DCV ainda não pode ser determinado. Entretanto, com frequência as DCV são intimamente relacionadas à aterosclerose, e alguns estudos anteriores demonstraram que uma concentração de AUS alta acelera seu desenvolvimento.^23 , 24^ Um grande estudo anterior da relação entre AUS e a aterosclerose precoce demonstrou que, em comparação com o quartil de AUS mais baixo, a espessura íntima-média da carótida (EIMC) era 37% mais alta em homens e 48% mais alta em mulheres no quartil de AUS mais alto.^25^ Vários possíveis mecanismos podem explicar a relação entre AUS e EIMC alta e DCV. Primeiramente, uma concentração alta de AUS pode levar à disfunção endotelial, e, portanto, ao desenvolvimento de DCV. Alguns estudos anteriores detectaram que reduções de AUS induzidas por alopurinol estão fortemente relacionadas à melhoria da função endotelial.^26^ Em experimentos com animais, identificou-se que um nível de AUS alto pode causar disfunção endotelial por meio de redução da produção de óxido nítrico.^27 - 29^ Segundo, o AUS pode induzir o aumento da secreção de substâncias pró-inflamatórias, levando a aumentos na inflamação vascular e na aterosclerose.^23 , 30 , 31^ Em terceiro lugar, um estudo longitudinal realizado no Instituto Nacional de Saúde demonstrou uma correlação positiva entre o AUS e a velocidade da onda de pulso em homens, o que pode ter sido mediado por meio de um efeito pró-inflamatório e/ou pela indução da proliferação de células do músculo liso.^32^ Portanto, o AUS alto pode não somente ter efeitos indiretos no avanço da aterosclerose, por sua associação com obesidade, hipertensão, diabetes, síndrome metabólica e outros fatores de risco cardiovascular convencionais, como também aumentar a incidência e a mortalidade associada a eventos cardiovasculares.^33 , 34^

No presente estudo, uma correlação positiva mais forte foi demonstrada entre a concentração de AUS e a incidência de DCV nos participantes com idade ≥40 anos. Isso pode ser mediado pelo efeito de exposição em longo prazo à hiperuricemia no avanço da aterosclerose. Vale ressaltar que, no presente estudo, em comparação com participantes no Q1, os homens e mulheres no Q4, com concentrações médias de AUS de 384,4 μmol/L e 290,5 μmol/L, respectivamente, que ficam abaixo dos critérios diagnósticos de hiperuricemia, ainda assim, tinham riscos significativamente mais altos de DCV. Portanto, deve-se estar atento para o caso de pacientes que possam ter concentrações de AUS normais-altas. Notadamente, a relação entre AUS e a incidência de DCV estava presente em participantes com IMC e CC normais. Os mecanismos subjacentes ainda não estão claros, mas, considerando que a obesidade é um fator de risco importante de DCV, ela ainda pode ocultar um efeito do AUS alto na incidência de DCV em uma população obesa.

O presente estudo tem algumas limitações. O AUS foi medido somente uma vez, na linha de base, como em quase todos os estudos anteriores, e, portanto, não se pode excluir a possibilidade de que alguns desses participantes tenham tido apenas um aumento temporário de AUS no momento do cadastro. Foi estudada uma população residente nas cidades do norte da China que trabalha em universidades, hospitais, órgãos governamentais, ou empresas, e, portanto, com grau de escolaridade alto e renda estável. Dessa forma, não se pode ter certeza de que esses resultados poderiam ser extrapolados para outras regiões. Portanto, é necessário que se realizem estudos posteriores em grande escala e multicêntricos. Além disso, embora uma variedade de possíveis fatores de confusão tenham sido padronizadas, ainda existe uma possibilidade de fatores de confusão residuais, tais como exercícios diários, dieta, stress e histórico familiar, que não foram avaliados no presente estudo. Entretanto, apesar das limitações acima, acredita-se que nossos achados fazem uma contribuição valiosa ao conhecimento da ligação entre AUS e a incidência de DCV.

## Conclusão

Em resumo, o presente estudo confirmou que a alta concentração de AUS é um fator de risco independente para DCV em pessoas de meia-idade e idosos. A hiperuricemia deve ser considerada um risco potencial e levada em consideração como alternativa para a prevenção e o tratamento de DCV.

## References

[1] Qiu L, Cheng XQ, Wu J, Liu JT, Xu T, Ding HT, et al. Prevalence of hyperuricemia and its related risk factors in healthy adults from Northern and Northeastern Chinese provinces. BMC Public Health. 2013 Jul 17;13:664. 10.1186/1471-2458-13-664 PMC3722003 23866159

[2] Liu H, Zhang XM, Wang YL, Liu BC. Prevalence of hyperuricemia among Chinese adults: a national cross-sectional survey using multistage, stratified sampling. J Nephrol. 2014;27(6):653-8. 10.1007/s40620-014-0082-z 24687401

[3] Kuwabara M, Kuwabara R, Hisatome I, Niwa K, Roncal-Jimenez CA, Bjornstad P, et al. “Metabolically Healthy” obesity and hyperuricemia increase risk for hypertension and diabetes: 5-year Japanese Cohort Study. Obesity (Silver Spring). 2017;25(11):1997-2008. 10.1002/oby.22000 PMC5846469 28922565

[4] Yokoi Y, Kondo T, Okumura N, Shimokata K, Osugi S, Maeda K, et al. Serum uric acid as a predictor of future hypertension: Stratified analysis based on body mass index and age. Prev Med. 2016 Sep;90:201-6. 10.1016/j.ypmed.2016.07.007 27404578

[5] Bener A, Al-Hamaq A, Öztürk M, Tewfik I. Vitamin D and elevated serum uric acid as novel predictors and prognostic markers for type 2 diabetes mellitus. J Pharm Bioallied Sci. 2019;11(2):127-32. 10.4103/jpbs.JPBS_240_18 PMC6537638 31148888

[6] Guan T, Ma J, Li M, Xue T, Lan Z, Guo J, et al. Rapid transitions in the epidemiology of stroke and its risk factors in China from 2002 to 2013. Neurology. 2017;89(1):53-61. 10.1212/WNL.0000000000004056 28566547

[7] Yang G, Wang Y, Zeng Y, Gao GF, Liang X, Zhou M, et al. Rapid health transition in China, 1990-2010: findings from the Global Burden of Disease Study 2010. Lancet. 2013;381(9882):1987-2015. 10.1016/S0140-6736(13)61097-1 PMC7159289 23746901

[8] Culleton BF, Larson MG, Kannel WB, Levy D. Serum uric acid and risk for cardiovascular disease and death: the Framingham Heart Study. Ann Intern Med. 1999;131(1):7-13. 10.7326/0003-4819-131-1-199907060-00003 10391820

[9] Lai X, Yang L, Légaré S, Angileri F, Chen X, Fang Q, et al. Dose-response relationship between serum uric acid levels and risk of incident coronary heart disease in the Dongfeng-Tongji Cohort. Int J Cardiol. 2016 Dec 1;224:299-304. 10.1016/j.ijcard.2016.09.035 27665401

[10] Kanellis J, Feig DI, Johnson RJ. Does asymptomatic hyperuricaemia contribute to the development of renal and cardiovascular disease? An old controversy renewed. Nephrology (Carlton). 2004;9(6):394-9. 10.1111/j.1440-1797.2004.00336.x 15663643

[11] Purnima S, El-Aal BG. Serum uric acid as prognostic marker of coronary heart disease (CHD). Clin Investig Arterioscler. 2016;28(5):216-24. 10.1016/j.arteri.2016.05.006 27663421

[12] Sun Y, Zhang H, Tian W, Shi L, Chen L, Li J, et al. Association between serum uric acid levels and coronary artery disease in different age and gender: a cross-sectional study. Aging Clin Exp Res. 2019;31(12):1783-90. 10.1007/s40520-019-01137-2 30694512

[13] Liu R, Han C, Wu D, Xi X, Gu J, Guan H, et al. Prevalence of hyperuricemia and gout in Mainland China from 2000 to 2014: a systematic review and meta-analysis. Biomed Res Int. 2015;2015:762820. 10.1155/2015/762820 PMC4657091 26640795

[14] Ma YC, Zuo L, Chen JH, Luo Q, Yu XQ, Li Y, et al. Modified glomerular filtration rate estimating equation for Chinese patients with chronic kidney disease. J Am Soc Nephrol. 2006;17(10):2937-44. 10.1681/ASN.2006040368 16988059

[15] Wei F, Sun N, Cai C, Feng S, Tian J, Shi W, et al. Associations between serum uric acid and the incidence of hypertension: a Chinese senior dynamic cohort study. J Transl Med. 2016;14(1):110. 10.1186/s12967-016-0866-0 PMC4851787 27129957

[16] Khera R, Lu Y, Lu J, Saxena A, Nasir K, Jiang L, et al. Impact of 2017 ACC/AHA guidelines on prevalence of hypertension and eligibility for antihypertensive treatment in United States and China: nationally representative cross sectional study. BMJ. 2018 Jul 11;362:k2357. 10.1136/bmj.k2357 PMC6039831 29997129

[17] Wei Y, Wang J, Han X, Yu C, Wanf F, Yuan J, et al. Metabolically healthy obesity increased diabetes incidence in a middle-aged and elderly Chinese population. Diabetes Metab Res Rev. 2020;36(1):e3202. 10.1002/dmrr.3202 31291052

[18] Kim SY, Guevara JP, Kim KM, Choi HK, Heitjan DF, Albert DA. Hyperuricemia and coronary heart disease: a systematic review and meta-analysis. Arthritis Care Res (Hoboken). 2010;62(2):170-80. 10.1002/acr.20065 PMC3156692 20191515

[19] Kim SY, Guevara JP, Kim KM, Choi HK, Heitjan DF, Albert DA. Hyperuricemia and risk of stroke: a systematic review and meta-analysis. Arthritis Rheum. 2009;61(7):885-92. 10.1002/art.24612 PMC2714267 19565556

[20] Wu J, Qiu L, Cheng XQ, Xu T, Wu W, Zeng XJ, et al. Hyperuricemia and clustering of cardiovascular risk factors in the Chinese adult population. Sci Rep. 2017;7(1):5456. 10.1038/s41598-017-05751-w PMC5511152 28710367

[21] Kuwabara M, Niwa K, Hisatome I, Nakagawa T, Roncal-Jimenez CA, Andres-Hernando A, et al. Asymptomatic hyperuricemia without comorbidities predicts cardiometabolic diseases: five-year Japanese Cohort Study. Hypertension. 2017;69(6):1036-44. 10.1161/HYPERTENSIONAHA.116.08998 PMC5426964 28396536

[22] Chuang SY, Chen JH, Yeh WT, Wu CC, Pan WH. Hyperuricemia and increased risk of ischemic heart disease in a large Chinese cohort. Int J Cardiol. 2012;154(3):316-21. 10.1016/j.ijcard.2011.06.055 21862159

[23] Wijnands JM, Boonen A, Dagnelie PC, Greevenbroek MMJ, Kallen CJH, Ferreira I, et al. The cross-sectional association between uric acid and atherosclerosis and the role of low-grade inflammation: the CODAM study. Rheumatology (Oxford). 2014;53(11):2053-62. 10.1093/rheumatology/keu239 24917566

[24] Cicero AFG, Salvi P, D’Addato S, Rosticci M, Borghi C, Brisighella Heart Study Group. Association between serum uric acid, hypertension, vascular stiffness and subclinical atherosclerosis: data from the Brisighella Heart Study. J Hypertens. 2014;32(1):57-64. 10.1097/HJH.0b013e328365b916 24309486

[25] Chen Y, Xu B, Sun W, Wang T, Xu Y, Xu M, et al. Impact of the serum uric acid level on subclinical atherosclerosis in middle-aged and elderly Chinese. J Atheroscler Thromb. 2015;22(8):823-32. 10.5551/jat.26260 25740202

[26] Doehner W, Schoene N, Rauchhaus M, Leyva-Leon F, Pavitt DV, Reaveley DA, et al. Effects of xanthine oxidase inhibition with allopurinol on endothelial function and peripheral blood flow in hyperuricemic patients with chronic heart failure: results from 2 placebo-controlled studies. Circulation. 2002;105(22):2619-24. 10.1161/01.cir.0000017502.58595.ed 12045167

[27] Mazzali M, Kanellis J, Han L, Feng L, Xia YY, Chen Q, et al. Hyperuricemia induces a primary renal arteriolopathy in rats by a blood pressure-independent mechanism. Am J Physiol Renal Physiol. 2002;282(6):F991-7. 10.1152/ajprenal.00283.2001 11997315

[28] Choi YJ, Yoon Y, Lee KY, Hien TT, Kang KW, Kim KC, et al. Uric acid induces endothelial dysfunction by vascular insulin resistance associated with the impairment of nitric oxide synthesis. FASEB J. 2014;28(7):3197-204. 10.1096/fj.13-247148 24652948

[29] Khosla UM, Zharikov S, Finch JL, Nakagawa T, Roncal C, Mu W, et al. Hyperuricemia induces endothelial dysfunction. Kidney Int. 2005;67(5):1739-42. 10.1111/j.1523-1755.2005.00273.x 15840020

[30] Prasad M, Matteson EL, Herrmann J, Gulat R, Rihal C, Lerman LO, et al. Uric acid is associated with inflammation, coronary microvascular dysfunction, and adverse outcomes in postmenopausal women. Hypertension. 2017;69(2):236-42. 10.1161/HYPERTENSIONAHA.116.08436 PMC5233565 27993955

[31] Valle M, Martos R, Cañete MD, Valle R, Donkelaar EL, Bermudo F, et al. Association of serum uric acid levels to inflammation biomarkers and endothelial dysfunction in obese prepubertal children. Pediatr Diabetes. 2015;16(6):441-7. 10.1111/pedi.12199 25131560

[32] Canepa M, Viazzi F, Strait JB, Ameri P, Pontremoli R, Brunelli C, et al. Longitudinal association between serum uric acid and arterial stiffness: results from the Baltimore Longitudinal Study of Aging. Hypertension. 2017;69(2):228-35. 10.1161/HYPERTENSIONAHA.116.08114 PMC5354105 27956574

[33] Kanbay M, Jensen T, Solak Y, Le M, Roncal-Jimenez C, Rivard C, et al. Uric acid in metabolic syndrome: from an innocent bystander to a central player. Eur J Intern Med. 2016 Apr;29:3-8. 10.1016/j.ejim.2015.11.026 PMC4826346 26703429

[34] Bombelli M, Quarti-Trevano F, Tadic M, Facchetti R, Cuspidi C, Mancia G, et al. Uric acid and risk of new-onset metabolic syndrome, impaired fasting glucose and diabetes mellitus in a general Italian population: data from the Pressioni Arteriose Monitorate E Loro Associazioni study. J Hypertens. 2018;36(7):1492-8. 10.1097/HJH.0000000000001721 29543626

