# Supplementary material for: Impacto da Concentração de Ácido Úrico Sérico no Risco de Doença Cardiovascular: Um Estudo Coorte Realizado no Norte da China
Source: Arq Bras Cardiol. 2021 Jul 7;117(4):666–75. [Article in Portuguese] doi: 10.36660/abc.20200378 (PMC8528365; doi:10.36660/abc.20200378)
Supplement: Translation

# Translation: Impact of Serum Uric Acid Concentration on the Risk of Cardiovascular Disease: A Cohort Study Conducted in Northern China

**Authors:** Qian Nie, Xuemei Zhang, Zhihua Hao, Liqin Wang, Huanxin Liu, Chenghao Liu, Zhongli Wang, Guangyao Song

**Affiliations:** 1 China Hebei Medical University - Department of Internal Medicine, Shijiazhuang City - China; 2 China Hebei General Hospital - Physical Examination Center, Shijiazhuang, Hebei - China; 3 China Hebei Medical University - School of Public Health, Shijiazhuang City, Hebei - China; 4 China Hebei General Hospital - Hebei Key Laboratory of Metabolic Diseases, Shijiazhuang - China

**Keywords:** Hyperuricemia, Uric Acid, Cardiovascular Diseases/incidence, Risk Factors, Body Weight

## Abstract

**Background:**

The results of previous studies of the relationship between serum uric acid (SUA) and the risk of cardiovascular disease (CVD) have been inconsistent due to confounding factors caused by other known cardiovascular risk factors.

**Objectives:**

This study aimed to evaluate the relationship between SUA and incident CVD in middle-aged and elderly Chinese people, who were stratified according to body mass index (BMI).

**Methods:**

This study recruited 5,721 participants of 40–75 years of age, who were free of CVD at baseline and who underwent follow-up from 2008 to 2017. Participants were categorized in SUA quintiles. Cox proportional hazard and Kaplan-Meier survival analysis were used to compare CVD incidence among the SUA groups. The correlations between SUA and CVD incidence in groups with differing BMI and waist circumference (WC) were also analyzed. A P value <0.05 was considered statistically significant.

**Results:**

During a mean follow-up period of 7.6 years, CVD incidence increased with SUA (log-rank test p<0. 001). Compared with the first quintile, the adjusted hazard ratios (95% confidence interval (CI)) for the development of CVD were 1.08 (0.78–1.65), 1.17 (0.88–1.77), 1.47 (1.12–2.21), and 1.68 (1.28–2.44) for the second to fifth quintiles, respectively. This relationship was clearer in participants with normal BMI and WC. The adjusted hazard ratio for each 100 μmol/L increase in SUA was 1.13 (95% CI: 1.02–1.39) for CVD events.

**Conclusions:**

High SUA is an independent risk factor for CVD in middle-aged and elderly northern Chinese people. This effect is maintained even after stratification according to measures of leanness/obesity.

## Introduction

Hyperuricemia has become highly prevalent in recent years, most likely as a result of rapid economic development and changes in lifestyle.^1 , 2^ Some previous studies have shown that high serum uric acid (SUA) concentration is associated with higher incidences of conventional risk factors for cardiovascular disease (CVD), such as hypertension, diabetes, and arteriosclerosis.^3 - 5^ CVDs, including coronary artery disease, heart failure, and stroke, are well-known to be the most common causes of morbidity and mortality worldwide.^6 , 7^ In recent decades, increasing evidence has accumulated of a relationship between hyperuricemia and the occurrence and prognosis of CVD. In the Framingham heart study,^8^ a longitudinal observational study of 6,763 participants, the SUA concentration proved to have no causal role in the development of or death from coronary heart disease. However, other studies have shown that hyperuricemia increases the risk of cardiovascular and cerebrovascular disease and mortality.^9 - 11^ In a cross-sectional study conducted in northeast China,^12^ SUA was associated with coronary artery disease in women, and particularly in those who were >80 years old, but not in men. These contradictory findings may be explained by confounding factors caused by numerous other risk factors.

Hyperuricemia is common in the Chinese population, especially in economically developed regions.^13^ Therefore, it is important for public health that the status of SUA as a risk factor for CVD and the SUA concentration that requires intervention should be determined. Therefore, the present study aimed to determine the relationship between SUA concentration and the risk of CVD in a cohort of middle-aged and elderly Chinese people over a 10-year period.

## Methods

### Ethical standards

All procedures performed in studies involving human participants were in accordance with the ethical standards of the institutional and/or national research committee and with the 1964 Helsinki declaration and its later amendments or comparable ethical standards. The study was approved by the ethics committee of Hebei General Hospital (No. 190106). Informed consent was obtained from all the participants included in the study.

### Study population

This cohort study recruited 40–75-year-old participants in comprehensive annual health screening at Hebei General Hospital, Shijiazhuang, China in 2008. These participants were employed by companies, universities, local governmental organizations, and hospitals. At the follow-up investigation until 2017, participants repeated the health screening each year, comprised of a questionnaire interview, physical examinations, and blood collection, similar to those during the baseline survey. This study was approved by the Human Ethics Committee of Hebei General Hospital (No.190106). Written informed consent was obtained from each participant.

For the analysis, CVD was defined as an ischemic heart disease (IHD), hemorrhagic stroke, or ischemic stroke. IHD was defined as myocardial infarction, angina pectoris, percutaneous coronary intervention, or coronary artery bypass surgery. The cohort consisted of 11,842 participants who had not experienced IHD, stroke, cancer, or kidney failure prior to enrollment, from 2008, and who had undergone annual medical examinations until 2017. This study excluded 4,892 participants due to incomplete data, the presence of comorbidities, or a lack of lifestyle assessment in any year. Participants were excluded because they developed cancer (n=189), chronic nephritis or renal failure (n=106), or a CVD event within the first year of the study (n=68); because they died from causes other than CVD (n=45); or because they were lost to follow-up (n=821). After the exclusion of these participants, data from a total of 5,721 participants (3,156 men and 2,565 women) were analyzed ( Figure.1 ).

### Data collection using questionnaires

Each participant underwent an annual physical examination and completed a structured questionnaire regarding their general medical history, use of medication, history of surgery, and family medical history. Questions regarding smoking (never, former, or current smoker) and alcohol consumption (never, former, or current drinker) were included.

### Anthropometric measurements

Anthropometric data were collected annually, including height, body mass, waist circumference (WC), systolic blood pressure (SBP), and diastolic blood pressure (DBP), which were measured by well-trained nurses. The body mass index (BMI) was calculated as body mass in kilograms divided by the square of height in meters. Blood pressure was measured using a standard mercury sphygmomanometer (Omron HEM-7125, Dalian, China). The participants rested for >5 min before their blood pressure was measured. Height and body mass were measured while the participant was wearing light clothing and no shoes. These measurements were made twice during each physical examination, and the mean values were used in subsequent analyses.

### Laboratory assessment

Venous blood samples were collected from all of the participants, following a 12-h overnight fast, and were used to measure white blood cell (WBC) and platelet (PLT) counts; hemoglobin (Hb), serum uric acid (SUA), fasting plasma glucose (FPG), creatinine (Cr), triglyceride (TG), total cholesterol (TC), low-density lipoprotein-cholesterol (LDL-C), and high-density lipoprotein-cholesterol (HDL-C) concentrations; and alanine aminotransferase (ALT) and aspartate aminotransferase (AST) activities. All of these measurements were made using standard methods with an automated biochemistry analyzer (Beckman Coulter AU5800, Tokyo, Japan). Estimated glomerular filtration rate (eGFR) was calculated using the Modification of Diet in Renal Disease equation for Chinese patients with chronic kidney disease.^14^

### Definitions

Participants of each sex were separately allocated to five groups on the basis of their SUA concentration: <278 µmol/L, 278–324 µmol/L, 324–362 µmol/L, 362–417 µmol/L, and ≥417 µmol/L for men; and <210 µmol/L, 210–241 µmol/L, 241–272 µmol/L, 272–328 µmol/L, ≥ 328 µmol/L for women. Hyperuricemia was defined as an SUA ≥ 420 μmol/L in men and ≥ 360 μmol/L in women.^15^

Hypertension was defined as an SBP ≥ 140 mmHg and/or DBP ≥ 90 mmHg, or the current use of antihypertensive medication.^16^ Diabetes was defined as a physician-made diagnosis of diabetes reported by the participant, an FPG ≥ 7.0 mmol/L, or current use of antidiabetic medication.^9^ According to the recommended criteria for Chinese people, a BMI of 18.5–23.9 kg/m^2^was regarded as normal, a BMI of 24.0–27.9 kg/m^2^was regarded as indicating overweight, and a BMI ≥ 28 kg/m^2^was regarded as indicating obesity. Abdominal obesity was defined as a WC of >90 cm in men and of >85 cm in women.^17^ The primary endpoints were the development of IHD (International Classification of Disease-10 codes I20–25), hemorrhagic stroke (I61), or ischemic stroke (I63).

### Statistical analyses

The baseline characteristics of the participants were analyzed according to SUA quintile using descriptive statistics. Continuous variables are expressed as means ± standard deviations (SD) and categorical variables as percentages. The continuous variables were tested for normality through P-P plots. Comparisons between two groups were performed using unpaired Student’s *t* -test for normally distributed data and the chi-square test for categorical data. Multiple comparisons were performed using one-way ANOVA and the *P-* value was corrected by post hoc Bonferroni test. The Kaplan-Meier survival analysis and the log-rank test were used to compare the incidences of CVD among the SUA groups. Cox proportional hazards analysis was used to assess the relationship between SUA and the incidence of CVD. Statistical analyses were conducted using SPSS version 22.0 (IBM Inc., Armonk, NY, USA) for Windows. A p-value <0.05 was considered statistically significant.

## Results

### Baseline characteristic of the participants

The baseline characteristics of the cohort participants, categorized according to sex and SUA quintile, are shown in Tables 1 and 2 . BMI, WC, SBP, DBP, total cholesterol, triglycerides, LDL-C, ALT, Cr, FPG, and the prevalence of alcohol consumption, obesity, hypertension, and diabetes increased with higher serum uric acid levels in both sexes. Men with the highest SUA concentrations tended to be young, whereas women were older. In addition, a high SUA concentration was associated with low eGFR and HDL-C.

**Table 1:** – Baseline characteristics of the male participants, categorized according to quintile of serum uric acid concentration

	All	Q1	Q2	Q3	Q4	Q5	p
N (participants)	3156	635	631	632	631	627	-
SUA(μmol/L)	334.3±40.5	240.2±28.9	296.2±11.8	337.5±13.3	384.4±13.4	456.3±40.7	0.000
Age(years)	53.8±9.6	53.2±9.5	52.9±9.6	52.0±9.3	52.4±9.5	51.7±9.4	0.009
BMI(kg/m^2^)	25.6±2.9	24.5±3.2	25.4±2.8	25.7±2.8	26.2±2.7	26.9±2.6	0.000
WC(cm)	89.1±8.3	85.9±8.9	88.3±7.7	89.2±8.2	90.2±7,9	92.0±8.3	0.000
SBP(mmHg)	123.8±16.9	122.2±16.5	122.3±17.0	123.4±16.8	123.0±16.6	124.8±16.7	0.012
DBP(mmHg)	79.5±10.5	78.1±11.3	78.2±10.3	79.8±10.5	80.2±9.8	81.9±10.6	0.004
TC(mmol/L)	4.92±0.85	4.67±0.85	4.76±0.80	4.92±0.83	5.00±0.89	5.12±0.85	0.000
TG(mmol/L)	1.85±0.97	1.55±0.91	1.66±1.01	1.80±1.04	2.06±1.12	2.39±1.23	0.000
HDL-C(mmol/L)	1.27±0.27	1.32±0.28	1.26±0.24	1.25±0.26	1.24±0.25	1.23±0.27	0.000
LDL-C(mmol/L)	2.8±0.77	2.83±0.72	2.85±0.74	2.82±0.82	2.83±0.91	2.86±0.83	0.132
ALT(U/L)	25.0±11.8	23.3±10.5	23.4±10.8	25.4±11.5	28.2±11.3	28.7±11.9	0.000
AST(U/L)	22.7±8.5	22.2±9.3	21.9±6.7	23.1±10.7	23.8±10.2	23.9±6.8	0.097
Cr(μmol/L)	81.0±9.2	75.5±9.8	78.9±9.6	82.4±10.7	83.4±10.6	87.0±11.2	0.000
FPG(mmol/L)	5.96±1.05	5.31±1.81	5.96±0.89	5.97±0.91	6.02±1.11	6.05±0.85	0.000
eGFR(ml/min·1.73m^2^)	93.4±12.4	97.8±10.8	95.2±10.9	92.8±11.7	91.8±12.4	87.5±14.8	0.000
Current smokers (%)	40.67	40.39	42.77	41.83	39.98	41.48	0.326
Current drinkers (%)	37.23	35.69	36.66	38.02	41.86	42.78	0.000
Obesity (%)	20.8	13.72	16.08	19.44	24.42	31.82	0.000
Hypertension (%)	16.98	11.76	12.86	14.36	20.16	25.48	0.000
Diabetes (%)	11.08	7.42	8.38	9.79	9.98	13.23	0.000

*SUA: serum uric acid; BMI: body mass index; WC: waist circumference; SBP: systolic blood pressure; DBP diastolic blood pressure; TC: total cholesterol; TG: triglyceride; LDL-C: low-density lipoprotein-cholesterol; HDL-C: high-density lipoprotein-cholesterol; ALT: alanine aminotransferase; AST: aspartate aminotransferase; Cr: creatinine; FPG: fasting plasma glucose; eGFR: estimated glomerular filtration rate. Data are presented as means ± standard deviations (SD) if continuous and numbers (percentages) if categorical. Comparisons were performed using one-way ANOVA or the chi-square test for categorical data. The serum uric acid concentrations for each quintile were <278, 278–324, 324–362, 362–417, and ≥417 μmol/L.*

**Table 2:** – Baseline characteristics of the female participants, categorized according to quintile of serum uric acid concentration

	All	Q1	Q2	Q3	Q4	Q5	p
N (participants)	2565	515	513	513	514	510	-
SUA(μmol/L)	259.8±40.9	186.2 ±18.2	226.0±8.5	255.3±10.9	290.5±13.4	365.2±30.7	0.000
Age(years)	52.1±9.0	48.6±7.7	50.3±8.7	52.0±8.6	53.5±8.9	57.1±9.2	0.000
BMI(kg/m^2^)	24.2±3.1	22.8±2.6	23.7±3.0	24.4±3.3	24.7±3.2	25.5±3.1	0.000
WC(cm)	79.3±9.5	75.1±7.2	77.9±8.3	79.6±8.7	81.2±8.6	83.6±7.4	0.000
SBP(mmHg)	118.6±19.3	110.7±15.1	116.7±17.6	118.8±18.5	123.6±19.6	124.0±19.5	0.000
DBP(mmHg)	72.3±9.7	69.1±8.3	71.8±9.8	73.0±9.5	74.2±10.0	74.6±9.8	0.000
TC(mmol/L)	5.15±0.92	4.86±0.90	4.99±0.86	5.21±0.86	5.22±0.93	5.58±0.92	0.000
TG(mmol/L)	1.46±0.95	1.03±0.52	1.22±0.61	1.45±0.59	1.64±0.60	2.09±0.92	0.000
HDL-C(mmol/L)	1.56±0.33	1.61±0.32	1.60±0.31	1.57±0.32	1.50±0.33	1.48±0.36	0.006
LDL-C(mmol/L)	2.92±0.78	2.89±0.73	2.95±0.73	2.99±0.79	3.01±0.81	3.04±0.90	0.056
ALT(U/L)	16.3±10.5	17.9±8.6	17.9±10.3	19.0±8.6	20.6±11.6	21.9±9.3	0.013
AST(U/L)	21.6±10.0	21.3±9.8	20.6±6.9	21.4±7.1	22.3±9.5	22.9±6.0	0.087
Cr(μmol/L)	61.3±9.1	58.6±8,1	59.6±8.4	60.9±8.6	61.9±9.0	66.8±10.02	0.000
FPG(mmol/L)	5.81±1.00	5.61±0.77	5.76±0.91	5.87±1.05	6.00±1.15	6.18±1.00	0.000
eGFR(ml/min·1.73m^2^)	97.7±12.3	101.9±11.3	99.9±11.5	96.5±12.6	94.2±12.9	87.1±14.0	0.000
Current smoker (%)	4.06	3.72	4.08	3.93	4.70	4.16	0.079
Current drinkers (%)	4.73	2.53	5.22	4.01	4.85	6.08	0.000
Obesity (%)	14.54	3.80	7.23	13.71	16.36	17.51	0.000
Hypertension (%)	20.34	8.86	16.87	18.39	23.05	26.72	0.000
Diabetes (%)	8.78	2.11	6.02	6.45	10.41	11.30	0.000

*SUA: serum uric acid; BMI: body mass index; WC: waist circumference; SBP: systolic blood pressure; DBP diastolic blood pressure; TC: total cholesterol; TG: triglyceride; LDL-C: low-density lipoprotein-cholesterol; HDL-C: high-density lipoprotein-cholesterol; ALT: alanine aminotransferase; AST: aspartate aminotransferase; Cr: creatinine; FPG: fasting plasma glucose; eGFR: estimated glomerular filtration rate. Data are presented as means ± standard deviations (SD) if continuous and numbers (percentages) if categorical. Comparisons were performed using one-way ANOVA or the chi-square test for categorical data. The serum uric acid concentrations for each quintile were <210, 210–241, 241–272, 272–328, and ≥328 μmol/L.*

### The relationship between SUA and the risk of CVD

The baseline and follow-up SUA concentrations are compared for each sex in Figure 2 . SUA significantly increased from Q1 to Q5, both at baseline and during the follow-up period, according to ANOVA. The mean SUA concentration was lower at the end of the study than at baseline in men in Q5, but it still fulfilled the diagnostic criterion for hyperuricemia. The baseline characteristics of the participants, categorized according to incident CVD, are shown in Table 3 . The mean follow-up period was 7.6 years, during which the cumulative CVD incidence was 14.3% (n=821) among the participants, with incidences of 13.9% (n=438) in men and 14.9% (n=383) in women. In addition, compared with participants who did not develop CVD, those who did develop CVD had higher BMI, WC, SBP, DBP, TC, TG, LDL-C, ALT, and FPG. There was no significant difference in Cr between the two groups.

**Figure 2:** – Baseline and follow-up serum uric acid concentrations in participants of each sex. SUA, serum uric acid. Multiple comparisons were performed using one-way ANOVA and the P-value was corrected by post hoc Bonferroni test. In both men (A) and women (B), there were significant differences in SUA among the five groups at baseline and during follow-up. *P<0.05 (Q1 vs. other quintiles at baseline SUA concentrations), #P<0.05 (Q1 vs. other quintiles at follow-up SUA concentrations).

**Table 3:** – Baseline characteristics of the participants, categorized according to whether cardiovascular disease developed or not

	CVD (-)	CVD (+)	p
SUA (μmol/L)	299.4±75.1	319.5±73.6	0.012
Age (years)	52.2±9.2	59.6±9.1	0.000
Men (%)	47.1	53.4	–
BMI (kg/m^2^)	24.9±3.1	25.7±3.2	0.001
WC(cm)	84.4±9.7	87.4±9.5	0.000
SBP (mmHg)	120.9±18.1	131.9±19.0	0.000
DBP (mmHg)	76.1±10.7	77.8±10.6	0.037
TC(mmol/L)	5.0±0.8	5.3±0.9	0.001
TG(mmol/L)	1.6±0.7	2.0±1.0	0.000
HDL-C(mmol/L)	1.41±0.3	1.27±0.3	0.000
LDL-C(mmol/L)	2.86±0.7	3.1±0.8	0.034
Cr(μmol/L)	71.8±14.3	72.1±14.1	0.061
FPG (mmol/L)	5.9±1.0	6.4±1.2	0.000
**Smoker**			
Never (%)	67.3	58.4	
Former (%)	11.6	17.7	
Current (%)	21.0	23.8	0.000
**Alcohol intake**			
Never (%)	76.1	69.5	
Former (%)	3.2	8.6	
Current (%)	20.6	21.8	0.000

*SUA: serum uric acid; BMI: body mass index; WC: waist circumference; SBP: systolic blood pressure; DBP diastolic blood pressure; TC: total cholesterol; TG: triglyceride; LDL-C: low-density lipoprotein-cholesterol; HDL-C: high-density lipoprotein-cholesterol; Cr: creatinine; FPG: fasting plasma glucose. Comparisons were performed using Student’s t-test for continuous variables and the Chi-square test for categorical data. Data was presented as means ± standard deviations (SD) for continuous variable and numbers (percentage) for category variables.*

As shown in Figure 3A , Kaplan-Meier analysis of CVD incidence in the SUA quintiles over the 10 years of the study showed significant differences between each group (log-rank test p < 0.001). Increases in SUA were associated with higher incidences of CVD. Because SUA is affected by renal function, we next categorized the participants according to eGFR and reanalyzed the data, and found that the relationship between SUA and CVD remained intact in participants who had eGFR values within the normal range (log-rank test p < 0.001; Figure 3B ).

**Figure 3:** – *Kaplan-Meier curves for incident cardiovascular disease for each quintile of serum uric acid (SUA) concentration. (A) All participants (log-rank test p-value < 0.01, Q1 vs. other quintiles). (B) Participants with normal eGFR (log-rank test p-value < 0.01, Q1 vs. other quintiles).*

Cox proportional hazards analysis was used to further assess the relationship between SUA concentration and CVD incidence ( Table 4 ). In the crude model, compared with SUA Q1, the hazard ratios (HRs) for CVD in the other four SUA quintiles were 1.18 (0.82–1.70), 1.41 (1.01–1.98), 1.95 (1.40–2.71), and 2.58 (1.84–3.61), respectively. The risks remained significantly different after adjustment for age in model 2, and age and sex in model 3. The HR decreased gradually as the number of adjusted covariates increased, but the association between SUA and CVD remained significant after adjustment for age, sex, eGFR, BMI, BP, total cholesterol, triglyceride, FPG, smoking, and alcohol intake (model 4). Compared with Q1, the HRs (95% CIs) for Q2–Q5 were 1.08 (0.78–1.65), 1.17 (0.88–1.77), 1.47 (1.12–2.21), and 1.68 (1.28–2.44). The adjusted HR for each 100 μmol/L increase in SUA was 1.13 (95% CI: 1.02–1.39) for CVD events. We subsequently reanalyzed the data after stratification according to BMI and WC, and found that the significant association between SUA and CVD was more evident in participants with normal BMI and WC ( Table 5 ).

**Table 4:** – Hazard ratios for incident cardiovascular disease

	Number of events	Hazard ratio (95% CI)			
	
SUA levels		Model 1	Model 2	Model 3	Model 4
Q1	106/1150	1	1	1	1
Q2	124/1144	1.18(0.82-1.70)	1.56(0.80-1.66)	1.12(0.77-1.63)	1.08(0.78-1.65)
Q3	146/1145	1.41(1.01-1.98)	1.34(0.96-1.89)	1.27(0.91-1.79)	1.17(0.88-1.77)
Q4	203/1145	1.95(1.40-2.71)	1.73(1.25-2.41)	1.61(1.15-2.23)	1.47(1.12-2.21)
Q5	242/1137	2.58(1.84-3.61)	2.08(1.48-2.91)	1.89(1.34-1.34)	1.68(1.28-2.44)
SUA per 100 μmol/L		1.25(1.11-1.42)	1.18(1.04-1.34)	1.16(1.09-1.29)	1.13(1.02-1.39)

*CI: confidence interval; SUA: serum uric acid. Model 1 used the crude data. Model 2 was adjusted for age. Model 3 was adjusted for age and sex. Model 4 was adjusted for age, sex, eGFR, body mass index, blood pressure, total cholesterol, triglyceride, fasting plasma glucose, smoking, and alcohol intake.*

**Table 5:** – Adjusted hazard ratios for the risk of CVD associated with serum uric acid concentration, stratified according to body mass index and waist circumference

	Hazards radio(95%CI)				

	Q1	Q2	Q3	Q4	Q5
BMI (≤23.9)	Reference	1.15(0.92-1.29)	1.21(0.98-1.56)	1.26(1.01-1.64)	1.32(1.08-1.82)
BMI (24.0-27.9)	Reference	1.08(0.78-1.67)	1.13(0.69-1.89)	1.15(0.87-1.65)	1.18(0.98-1.83)
BMI (≥28)	Reference	0.92(0.74-1.56)	1.01(0.85-1.85)	0.98(0.78-1.49)	1.08(0.95-1.77)
WC (male<90,female<85)	Reference	0.89(0.78-1.45)	1.08(0.88-1.37)	1.12(1.01-1.43)	1.21(1.03-1.59)
WC (male≥90,female≥85)	Reference	1.02(0.72-1.78)	1.15(0.83-1.85)	1.23(0.75-1.91)	1.30(0.91-1.89)

*The adjusted hazard ratios were calculated in Model 4 and were stratified according to body mass index (BMI, kg/m2) and waist circumference (WC, cm). SUA Q1 was used as the reference group. The covariates used in Model 4 were age, sex, eGFR, body mass index, blood pressure, total cholesterol, triglyceride, fasting plasma glucose, smoking, and alcohol intake.*

## Discussion

The present cohort study found a significant positive relationship between SUA concentration and the CVD incidence in middle-aged and elderly Chinese people. This relationship was regardless of the potential confounding factors of age, BMI, SBP, DBP, TC, TG, HDL-C, LDL-C, FBG, and a history of hypertension or diabetes. In addition, our study showed that a 100 μmol/L increase in SUA increases the risk of CVD by 13%. These results demonstrate that SUA concentration is an independent risk factor for CVD.

Previous studies have also shown a relationship between SUA and CVD in other countries and regions. One previous meta-analysis showed that hyperuricemia increases the risk of CVD: the risk of CVD was 2.1-fold higher in people with a high SUA than in those with a low SUA.^18^ Another meta-analysis of 16 cohort studies showed that hyperuricemia is associated with a higher risk of stroke (risk ratio: 1.41, 95% CI: 1.05–1.76) and higher stroke-related mortality (risk ratio: 1.36, 95% CI: 1.03–1.69), and these relationships remained significant after adjustment for other risk factors, such as sex, age, and the presence of hypertension or diabetes.^19^ There is also an increasing amount of evidence that hyperuricemia increases the risk of cardiovascular and cerebrovascular disease and mortality.^9 , 11 , 20 - 22^ In the present study, the participants were middle-aged and elderly people living in northern cities in China, but the findings were consistent with and extend previous findings.

However, most of the previous studies focused on the relationship between SUA and CVD-related mortality, and only a few focused on the relationship with CVD incidence. It is worth noting that a 10-year cohort study of 128,569 people conducted in Taiwan^22^ showed that hyperuricemia is a potential risk factor for IHD. However, a weakness of this study was that SUA was divided into only high or normal groups, and the relationship was only adjusted for a history of hypertension or diabetes, smoking, alcohol consumption, and the use of diuretics. Thus, other important covariates, such as BMI, age, and eGFR were not adjusted for. The present study aimed to remedy these deficiencies by using quintiles of SUA to demonstrate the relationship between SUA and CVD after adjustment for a wide range of conventional risk factors. Furthermore, this relationship remained when SUA was treated as a continuous variable. Previous studies have also demonstrated a continuous relationship between SUA and CVD,^9^ and in the present study, the SUA values in Q4, which did not exceed the reference range, were associated with a significantly higher risk of CVD.

The mechanism by which a high SUA may increase the risk of CVD remains elusive. However, CVD is often closely related to atherosclerosis, and some previous studies have shown that a high SUA concentration accelerates its development.^23 , 24^ A large previous study of the relationship between SUA and early atherosclerosis showed that, compared with people in the lowest quartile of SUA, the carotid intima-media thickness (CIMT) was 37% higher in men and 48% higher in women in the highest SUA quartile.^25^ Several potential mechanisms may account for the relationship between SUA and high CIMT and CVD. First, a high SUA concentration may promote endothelial dysfunction, and therefore the development of CVD. Some previous studies have found that allopurinol-induced reductions in SUA correlate strongly with improvements in endothelial function.^26^ In animal experiments, it was found that high SUA may cause endothelial dysfunction by means of a reduction in nitric oxide production.^27 - 29^ Second, SUA may induce greater secretion of proinflammatory substances, leading to increases in vascular inflammation and atherosclerosis.^23 , 30 , 31^ Third, a longitudinal study conducted at the National Institutes of Health showed a positive correlation between SUA and pulse wave velocity in men, which may have been mediated though a pro-inflammatory effect and/or by inducing the proliferation of smooth muscle cells.^32^ Thus, high SUA may not only have indirect effects on the progression of atherosclerosis, through its association with obesity, hypertension, diabetes, the metabolic syndrome, and other conventional cardiovascular risk factors, but may also increase the incidence and mortality associated with cardiovascular events.^33 , 34^

In the present study, a stronger positive correlation was shown between SUA concentration and the CVD incidence in participants aged ≥40 years. This may be mediated though the effect of long-term exposure to hyperuricemia in the progression of atherosclerosis. It is worth noting that, in the present study, compared with participants in Q1, men and women in Q4, with mean SUA concentrations of 384.4 μmol/L and 290.5 μmol/L, respectively, which are below the diagnostic criteria for hyperuricemia, still presented significantly higher risks of CVD. Therefore, we should be vigilant if patients have high-normal SUA concentrations. Notably, the relationship between SUA and the CVD incidence was present in participants with normal BMI and WC. The underlying mechanisms are unclear, but given that obesity is an important risk factor for CVD, it may also conceal an effect of high SUA on the CVD incidence in an obese population.

There were some limitations to the present study. SUA was only measured once, at baseline, as in almost all of the previous studies, so we could not exclude the possibility that some of the participants may have had only a brief increase in SUA at the time of enrollment. This study analyzed a population living in the northern cities of China who work in universities, hospitals, government departments, or companies, and therefore had a high educational level and a stable income. Therefore, it is uncertain whether the results could be extrapolated to other regions. Thus, further large-scale, multi-center studies are required. Furthermore, although a variety of potential confounding factors were adjusted for, there remains a possibility of residual confounding due to factors such as daily exercise, diet, stress, and family history, which were not evaluated in the present study. However, despite the above limitations, we believe that our findings make a valuable contribution to knowledge of the link between SUA and the incidence of CVD.

## Conclusion

In summary, the present study confirmed that high SUA concentration is an independent risk factor for CVD in middle-aged and elderly people. Hyperuricemia should be considered as a potential risk and considered in the prevention and treatment of CVD.

**Figure 1:** – *Description of the study population. Participants of each sex were separately allocated to five groups (Q1-Q5) on the basis of their SUA concentration.*
